# Antioxidant effect of endothelin-1 receptor antagonist protects the rat kidney against chronic injury induced by hypertension and hyperglycemia

**DOI:** 10.1590/2175-8239-JBN-2018-0162

**Published:** 2019-09-02

**Authors:** Agnaldo Caires, Marcia Bastos Convento, Bianca Castino, Ala Moana Leme, Edson de Andrade Pessoa, Alef Aragão, Nestor Schor, Fernanda Teixeira Borges

**Affiliations:** 1 Universidade Federal de São Paulo, Departamento de Medicina, Disciplina de Nefrologia, São Paulo, SP, Brasil.; 2 Universidade Cruzeiro do Sul, Programa Interdisciplinar em Ciências da Saúde, São Paulo, SP, Brasil.

**Keywords:** Rats, Renal Insufficiency, Chronic, Endothelin-1, Antioxidants, Ratos, Insuficiência Renal Crônica, Endotelina-1, Antioxidantes

## Abstract

Hypertension and Diabetes mellitus are the two main causes of chronic kidney disease that culminate in the final stage of kidney disease. Since these two risk factors are common and can overlap, new approaches to prevent or treat them are needed. Macitentan (MAC) is a new non-selective antagonist of the endothelin-1 (ET-1) receptor. This study aimed to evaluate the effect of chronic blockade of ET-1 receptor with MAC on the alteration of renal function observed in hypertensive and hyperglycemic animals. Genetically hypertensive rats were divided into control hypertensive (HT-CTL) group, hypertensive and hyperglycemic (HT+DIAB) group, and hypertensive and hyperglycemic group that received 25 mg/kg macitentan (HT-DIAB+MAC25) via gavage for 60 days. Kidney function and parameters associated with oxidative and nitrosative stress were evaluated. Immunohistochemistry for neutrophil gelatinase-associated lipocalin (NGAL), ET-1, and catalase in the renal cortex was performed. The HT+DIAB group showed a decrease in kidney function and an increase in NGAL expression in the renal cortex, as well as an increase in oxidative stress. MAC treatment was associated with attenuated ET-1 and NGAL production and increases in antioxidant defense (catalase expression) and nitric oxide production. In addition, MAC prevented an increase in oxidant injury (as measured by urinary hydroperoxide and lipid peroxidation), thus improving renal function. Our results suggest that the antioxidant effect of the ET-1 receptor antagonist MAC is involved in the improvement of kidney function observed in hypertensive and hyperglycemic rats.

## INTRODUCTION

Diabetes mellitus (DM) and hypertension are the main causes of chronic kidney disease (CKD) and its progression to end-stage renal disease^1^. The incidence of CKD increases with age in both developed and developing countries^2^^,^ 3. Therefore, new strategies to prevent the development of CKD are necessary.

One common model of type 2 DM is the induction in rats through with a single injection of low-dose streptozotocin^4^. Spontaneously hypertensive rats (SHR) are an animal model frequently used to study essential (or primary) genetic hypertension^5^. The induction of diabetes in the SHR model^6^ accelerates the development of diabetic nephropathy and chronic kidney disease (CKD).

Diabetic nephropathy (DN) is a strong risk factor for vascular disease, common among patients with type 2 DM, and characterized by podocyte injury, proteinuria, glomerular fibrosis, and a decrease in glomerular filtration^7^. Features of DN include hyperglycemia, inflammation, reactive oxygen species (ROS) production, and endothelin-1 (ET-1) and renin-angiotensin system (RAS) activation^8^.

Untreated, diabetic nephropathy is the leading cause of end-stage chronic kidney disease^9^. CKD is characterized by an increase in kidney injury markers (urine albumin > 30 mg/24 h or albumin/creatinine ratio higher than 30 mg/g), tubular damage and impaired ionic excretion, abnormal histologic findings on kidney biopsy specimens, and decrease in the glomerular filtration rate (< 60 mL/min/1.73 m^2^), among other findings^10^.

ROS are involved in the pathophysiology of CKD, and NADPH oxidases are the main sources of ROS in many tissues^11^^-^ 13.

ET-1 modulates renal blood flow by regulating the vascular tone^14^. It is produced by endothelial cells, but under pathophysiological conditions, it can be produced by podocytes, and mesangial and parietal epithelial cells^15^. ET-1 exerts its function in target cells via two receptors, ETA and ETB^15^.

ETA receptor activation mediates vasoconstriction, vascular cell proliferation, and proteinuria^16^. Differently, ETB receptor activation releases vasodilating substances such as nitric oxide and prostacyclin, leading to vasodilation first and then vasoconstriction, stimulates sodium reabsorption in collecting ducts, and development of proteinuria^17^. Circulating ET-1 levels are increased in hypertensive patients^18^.

ET-1 and the RAS interact in renal tissues. Angiotensin II increases ET-1 production and upregulates ETA receptor expression in the kidney^19^^,^ 20. Together, both peptides can produce pre- and post-glomerular vasoconstriction, mesangial cell contraction, glomerular filtration rate decrease, extracellular matrix production and increase in tubular sodium reabsorption^21^. It is reasonable to propose that ET-1 receptor blockade may be beneficial in diabetic nephropathy.

Indeed, clinical trials to study the effect of the ET-1 antagonist atrasentan in DN are documented^22^. Currently, however, the principal therapeutic use of ET-1 receptor antagonists is in the treatment of idiopathic pulmonary fibrosis and pulmonary arterial hypertension^23^^,^ 24. For the first time, the present study evaluates the effect of macitentan, a new non-selective ET-1 receptor antagonist used in the treatment of pulmonary hypertension, on the progression of kidney injury observed in hypertensive and hyperglycemic rats.

## METHODS

### ANIMAL TREATMENT

The experimental protocol was approved by the Ethics Committee (CEP 798483) of the Universidade Federal de São Paulo (UNIFESP) and was performed in accordance with the Brazilian guidelines for scientific animal care and use^25^^,^ 26. Animals were obtained from the Centro de Desenvolvimento de Modelos Experimentais para Medicina e Biologia (CEDEME) of UNIFESP.

Thirty genetically hypertensive (SHR) male rats, aged 6 weeks and weighing 170-200 g, were maintained in metabolic cages. The cages were kept in a temperature (21 ± 2ºC) and humidity-controlled (60 ± 10%) room with a 12-h dark/light (artificial lights, 7 a.m.-7 p.m.) cycle and hourly air exhaust (15 min/h).

After an adaptation period of two weeks, the animals were divided into three groups, and during the 60-day study, all animals were fed standard rat chow *ad libitum* with free access to water.

Ten genetically hypertensive (HT) rats that were maintained in the control (CTL) situation received one intravenous injection of distilled water (vehicle only) and the group was named HT-CTL.

Twenty rats received one intravenous injection of 60 mg/kg streptozotocin (Sigma-Aldrich, Saint Louis, USA) in citrate buffer 0.1 M, pH 4.4), and only rats with blood glucose higher than 400 mg/dL were selected. Half of these were maintained in the HT and hyperglycemic (DIAB) group, and the group was named HT-DIAB. The remaining 10 rats were treated with daily macitentan (MAC) with 25 mg/kg administration by gavage for 60 days and the group was named HT-DIAB+MAC25.

At 0 (basal), 30, and 60 days after the beginning of experimental protocols, all animals were weighed, blood samples were collected from the lateral tail vein, and rats were maintained in metabolic cages for 24 h for urine collection, and the urine volume was quantified. The animals were euthanized 60 days after the beginning of the experimental protocol with a toxic ip dose of ketamine (90 mg/kg)/xylazine (10 mg/kg), (Agribands de Brasil Ltda, SP, Brazil). The right and left kidneys were then removed for immunohistochemical analysis. Biochemical parameters were measured in plasma and urine samples.

### MEASUREMENT OF BODY WEIGHT

The rats were weighed monthly using a 2610 scale (Labortex, SP, BRAZIL) and the result was reported in grams.

### MEASUREMENT OF SYSTOLIC BLOOD PRESSURE (MAP)

Systolic blood pressure was indirectly measured by tail plethysmography. Rats were placed in a warm chamber for 10 min, and the cuff and wrist receiver were attached to the tail. Blood pressure was recorded using an electric sphygmomanometer coupled to a 2-channel Gould model 2200 S polygraph (Record 2200S, Gould Inc., Cleveland, Ohio, USA). Measurements were taken at 0, 24, and 48 h, and the results are reported as means ± SD.

### BIOCHEMICAL ANALYSIS

The levels of plasma creatinine and plasma urea were assayed spectrophotometrically according to standard procedures, using commercially available diagnostic kits (Labtest Diagnostic, Brazil). Creatinine was determined by a colorimetric method based on the Jaffé reaction^27^. Urea was determined using a colorimetric assay based on urease activity^28^. Plasma glucose concentration was determined using tail blood samples (Accuchek, Boehringer Mannheim, Indianapolis, Ind., USA). Levels of creatinine, urea, and glucose are reported in mg/dL.

Urine sodium concentrations were determined with a Micronal B462 flame photometer (Micronal, São Paulo, Brazil). Sodium excretion is reported as percentage of mEq/24 h. Urinary protein was quantified using a colorimetric method based on pyrogallol red-molybdate^29^. The results are reported as mg protein/24 h urine.

### OXIDATIVE STRESS STUDIES

To assess lipid peroxidation, levels of the peroxidation product malondialdehyde were determined by measuring thiobarbituric acid-reactive substances (TBARS)^30^. For the quantification, 0.4 mL of the urine sample diluted with 0.6 mL water was added to a reaction mixture consisting of 1.0 mL 17.5% trichloroacetic acid (TCA) and 1.0 mL 0.6% thiobarbituric acid to form a red compound. This mixture was heated in a water bath at 95ºC for 20 min; the solution was then removed from the water bath and cooled on ice, followed by the addition of 1.0 mL 70% TCA. The solution was homogenized and incubated for 20 min followed by spectrophotometric measurement at 534 nm (A = 1.56×10^5^ M/cm). The data are reported as nM/mg urinary creatinine.

Urinary peroxides were determined by the ferrous oxidation of xylenol orange version 2 (FOX-2) method^31^. Ferrous iron is oxidized to ferric iron by peroxides contained in the samples. Xylenol orange reagent shows high selectivity for the Fe^3+^ ion, producing a purplish-blue complex whose absorbance can be measured at 560 nm (A = 4.3×10^4^ M/cm). The following reagent was prepared: 90 mL methanol; 10 mL double distilled water; 100 µM xylenol orange; 4 mM butylated hydroxytoluene (BHT); 25 mM sulfuric acid and 250 µM ferrous ammonium sulfate. The urine sample (100 µL) was mixed with 900 µL of FOX-2 reagent, vortexed and incubated for 30 minutes at room temperature. Solutions were then centrifuged at 15,000 *g* for 10 min at 4ºC for the removal of protein residues. The absorbance at 560 nm was read against a blank. The data are reported as nmol/g urinary creatinine.

### NITRIC OXIDE DETERMINATION

Nitric oxide (NO) was determined by the Griess method^32^. A mixture of 1% sulfanilamide (in 5% H_3_PO_4_) and 0.1% naphthylethylenediamine solution (Sigma-Aldrich, Saint Louis, USA) was added to the urine samples, and the absorbance at 546 nm was measured using a GENESYS 2 spectrophotometer (Spectronic Instruments, Rochester, USA). Nitrite, one of the stable metabolites of NO, was then estimated from a standard curve constructed using NaNO_2_. The assessment of the urine creatinine was performed and used to normalize the NO concentrations. The data are reported as nM/mg urinary creatinine.

### IMMUNOHISTOCHEMISTRY

Kidneys were dissected along the non-hilar axis and fixed in 10% phosphate-buffered formalin (Erviegas, Brazil). Kidney sections were fixed with 4% buffered paraformaldehyde, embedded in paraffin (Erviegas, Brazil) and 4-µm thick sections were prepared. Kidney sections were deparaffinized and rehydrated. Endogenous peroxidase activity was blocked with 5% H_2_O_2_ in absolute methanol for 10 min at room temperature. To expose the antigens, kidney sections were boiled in a target retrieval solution [1 mmol/L tris(hydroxymethyl)aminomethane (Tris), pH 9.0, with 0.5 mM ethylene glycol tetraacetic acid (EGTA)] for 10 min. Nonspecific binding was prevented by incubating the sections in phosphate buffered saline (PBS) containing 1% bovine serum albumin (BSA), 0.05% saponin, and 0.2% gelatin. Sections were then incubated overnight at 4ºC with primary antibodies against ET-1 (1:200, rabbit anti-rat; ABCAM, MA, USA), neutrophil gelatinase-associated lipocalin (NGAL) (1:200, rabbit anti-rat; ABCAM, MA, USA) or catalase (1:200, rabbit anti-rat; ABCAM, MA, USA), for 18 hours at 4ºC. Sections were washed and incubated with appropriate streptavidin- peroxidase-conjugated secondary antibodies (Dako, Glostrup, Denmark) for 1 h at room temperature. The sites of antibody-antigen reactions were visualized by staining with 0.5% 3,3’-diaminobenzidine tetrachloride (Dako). Digital photomicrographs were taken through a Leica DM 1000 upright microscope connected to a workstation computer through the Leica DFC 310 FX, LAS 3.8 Microscope Camera (Leica, Switzerland). Ten photomicrographs along the renal cortex were taken, the light brown staining was quantified (LAS software, version 3.8) and averaged for each rat. The data are reported as percentage of stained area.

### WESTERN BLOTTING

The protein concentration was verified by the method of Lowry^33^. Cells and kidney tissues were lysed with a 200-µL RIPA lysis buffer per plate (100 mm^2^). The lysates were centrifuged at 12,000 *g* for 5 min at 4ºC, and the supernatants were stored at −80ºC. Proteins (30 µg) were separated by 10% polyacrylamide gel electrophoresis and transferred to polyvinylidene fluoride (PVDF) membranes using a Mini Trans-Blot Electrophoretic Transfer Cell (BioRad, CA, USA). Nonspecific binding sites were blocked with 5% albumin (v/v) in TBS buffer. The immunoblots were incubated overnight at 4ºC with renin (1:500, Santa Cruz, TX, USA), angiotensin I (1:500, Santa Cruz, TX, USA), angiotensin II (1:500, Santa Cruz, TX, USA), or GAPDH (1:500, Abcam, MA, USA) primary antibodies. After washing three times with TBS-T, the membranes were incubated for 1 h at 4ºC in HRP-conjugated secondary antibodies (1:100,000; Santa Cruz TX, USA). Immunoreactive protein bands were visualized using Pierce ECL Plus Chemiluminescent substrate (Thermo Fisher, USA). Images were obtained and analyzed with an Alliance 7 Chemiluminescence documentation system (UVITEC, Cambridge, UK). The immunoblot band intensities were quantified using Image J software and reported as the renin/GAPDH, Angiotensin I/GAPDH, and Angiotensin II/GAPDH ratio.

### STATISTICAL ANALYSIS

Results are reported as a means ± SE. Data were analyzed by two-way analysis of variance (ANOVA) followed by the Tukey’s post-hoc test, and *p* < 0.05 was considered statistically significant.

## RESULTS

Table 1 shows the mean body weight (g) of the groups during the experiment. The mean weight of the HT-DIAB and HT-DIAB+MAC25 groups was significantly lower than that of the CTL group after 30 days of study.

**Table 1:** Physiological parameters of genetically hypertensive (SHR) animals in the control group (HT-CTL), hypertensive and hyperglycemic animals (HT-DIAB), hypertensive and hyperglycemic animals treated with macitentan (HT-DIAB+MAC25) for basal, 30 and 60 days

Weight (g)	HT-CTL	HT-DIAB	HT-DIAB + MAC 25mg
BASAL	174,00 ± 10,19	167,38 ± 10,76	189,17 ± 13,38
30 DAYS	279,00 ± 18,46	184,75 ± 12,75 *	182,83 ± 17,74 *
60 DAYS	318,71 ± 19,49	191,25 ± 21,33 **	172,50 ± 19,42 **
MAP (mmHg)	HT-CTL	HT-DIAB	HT-DIAB+MAC 25 mg
BASAL	173,00 ± 5,67	178,00 ± 5,14	176,83 ± 4,28
30 DAYS	178,86 ± 4,61	187,88 ± 7,05	191,00 ± 7,30
60 DAYS	181,43 ± 2,76	190,50 ± 6,70	195,17 ± 5,33
Plama Glucose (mg/dl)	HT-CTL	HT-DIAB	HT-DIAB + MAC 25 mg
BASAL	100,29 ± 8,29	100,29 ± 8,29	89,00 ± 0,82
30 DAYS	94,43 ± 2,17	418,50 ± 73,66 *	524,83 ± 7,21 *
60 DAYS	127,71 ± 14,11	541,38 ± 18,67 **	516,33 ± 30,79 **
Urine Volume (ml)	HT-CTL	HT-DIAB	HT-DIAB + MAC 25 mg
BASAL	11,11 ± 1,41	9,12 ± 0,84	12,83 ± 0,91
30 DAYS	13,05 ± 1,57	76,83 ± 8,98 *	78,33 ± 7,03 *
60 DAYS	13,91 ± 1,69	85,00 ± 5,80 **	78,33 ± 9,80 **
Plasma Creatinine (mg/dl)	HT-CTL	HT-DIAB	HT-DIAB + MAC
BASAL	0,69 ± 0,07	0,61 ± 0,04	0,63 ± 0,05
30 DAYS	0,70 ± 0,06	0,85 ± 0,09	0,69 ± 0,05 ^¥^
60 DAYS	0,65 ± 0,03	0,98 ± 0,09 **	0,72 ± 0,01 ^¥¥^
Plasma Urea (mg/dl)	HT-CTL	HT-DIAB	HT-DIAB+MAC 25 mg
BASAL	26,59 ± 2,57	26,43 ± 3,22	23,00 ± 1,08
30 DAYS	38,86 ± 7,84	67,84 ± 11,12 *	55,75 ± 9,41 ^¥^
60 DAYS	36,53 ± 6,09	72,73 ± 11,04 **	47,25 ± 5,65
Urine Sodium (mEq/24h)	HT-CTL	HT-DIAB	HT-DIAB+MAC 25 mg
BASAL	10,61 ± 3,05	8,38 ± 2,35	14,11 ± 1,81
30 DAYS	12,97 ± 2,68	55,24 ± 3,28 *	56,46 ± 5,34 *
60 DAYS	10,61 ± 1,27	72,33 ± 14,10 **	44,42 ± 6,39 **
Urine Protein (mg/24h)	HT-CTL	HT-DIAB	HT-DIAB+MAC 25 mg
BASAL	8,79 ± 2,20	6,29 ± 1,21	10,04 ± 1,12
30 DAYS	12,85 ± 1,28	36,71 ± 4,89 *	29,81 ± 4,00 *
60 DAYS	10,97 ± 2,17	30,02 ± 3,58 **	21,40 ± 4,79 ** ^¥¥^

Data are reported as means ± SE. The significance level for a null hypothesis was set at 5% (*p* < 0.05).

(*) compared to the HT-CTL 30 days group;

(¥) compared to the HT-DIAB 30 days group,

(**) compared to the HT-CTL 60 days group, and

(¥¥) compared to the HT-DIAB 60 days group (ANOVA followed by the Tukey's post-hoc test). N = 10 per group.

There was no difference in the MAP in HT-DIAB and HT-DIAB+MAC25 groups when compared to the HT-CTL group within their respective experimental period. There was an increase in blood glucose in all experimental groups at 30 and 60 days when compared to their respective HT-CTL group.

All experimental groups exhibited an increase in urine production at 30 and 60 days when compared to their respective HT-CTL group.

The HT-DIAB group showed a significant increase in plasma creatinine and urea when compared to the HT-CTL group at 60 days. Notably, plasma creatinine and urea in the rats of the HT-DIAB+MAC25 group at 30 and 60 days were not statistically different when compared to their respective HT-CTL group.

The renal tubular function was evaluated by means of sodium excretion. In all experimental groups, we observed an increase in sodium excretion at 30 and 60 days when compared to their respective HT-CTL group. No statistical difference in sodium excretion was found between the diabetic rats treated or not with macitentan.

We observed a significant increase in urinary protein excretion in the HT-DIAB group in comparison to the HT-CTL group in all experimental periods. In the HT-DIAB+MAC25 group, we also observed an increase in protein excretion at 30 and 60 days when compared to their respective HT-CTL group. Nevertheless, diabetic rats treated with macitentan exhibited significantly decreased urinary protein excretion when compared to the HT-DIAB group at 60 days, suggesting improved renal barrier function.

**Figure 1:**
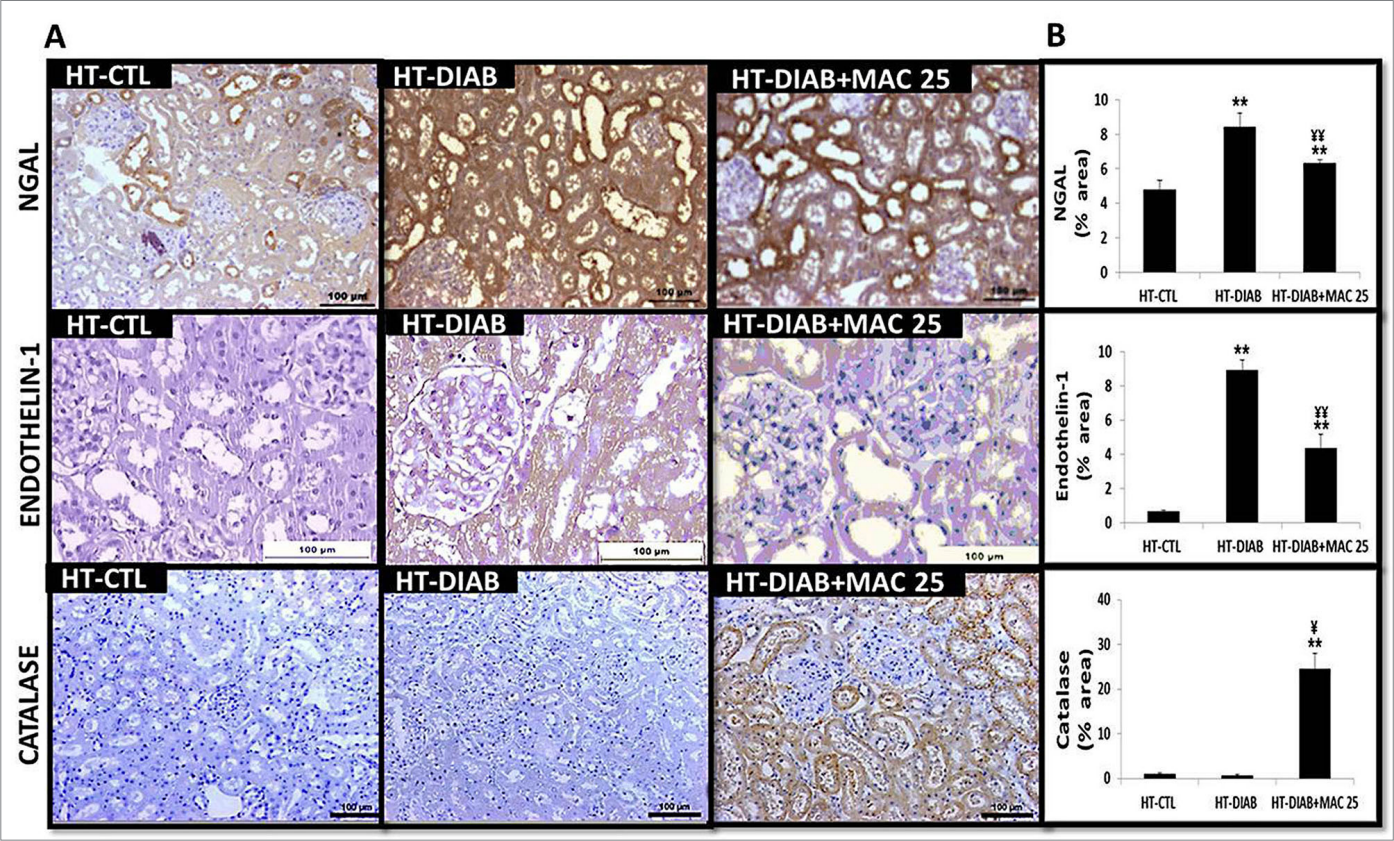
Light microscopy of immunostained kidney sections A, Neutrophil gelatinase-associated lipocalin (NGAL 200×), endothelin-1 (ET-1, 200×), and catalase (200×) in genetically hypertensive rats in the control group (HT-CTL), hypertensive and hyperglycemic animals (HT-DIAB), hypertensive and hyperglycemic animals treated with macitentan (HT-DIAB+MAC25) for 60 days (final day of the experiment). B, Quantitative analyses of kidney sections stained for NGAL, ET-1, and catalase. Data are reported as %. The significance level for a null hypothesis was set at 5%. ^**^ *p* < 0.05 compared to the HT-CTL 60 days group and ^¥¥^ *p* < 0.05 compared to the HT-DIAB 60 days group (ANOVA followed by the Tukey’s post-hoc test). N= 10 per group.

Immunostaining for ET-1 and NGAL at 60 days is shown in Figure 1. We observed an increase in the labeling of ET-1 and NGAL in the HT-DIAB group compared to the HT-CTL group, and a decrease when comparing the HT-DIAB+MAC25 group with the HT-DIAB group. MAC attenuated the increase in ET-1 and NGAL (a renal injury marker), corroborating the hypothesis of a beneficial effect on the kidney.

Figure 1 demonstrates the immunostaining for catalase at 60 days. We observed that the HT-DIAB group did not stimulate the expression of this antioxidant enzyme. However, the HT-DIAB+MAC25 group exhibited increase of catalase compared to the HT-CTL and HT-DIAB groups, suggesting an antioxidant potential for macitentan.

**Figure 2:**
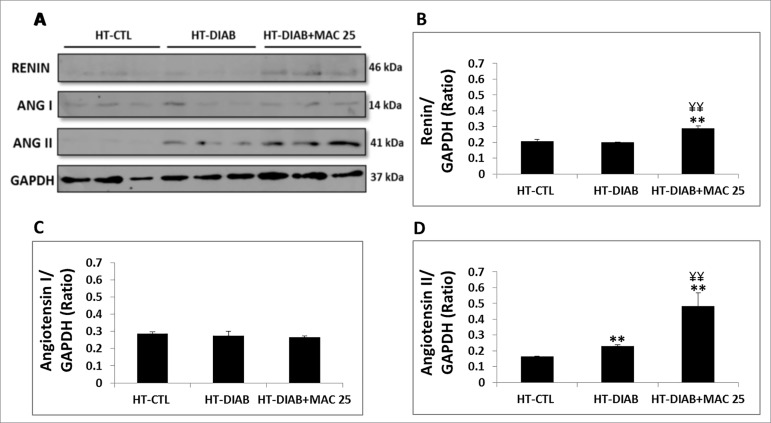
Renin, angiotensin I (ANG I), and angiotensin II (ANG II) analysis. A, Western blot image of kidney cortex of genetically hypertensive rats (SHR) in the control group (HT-CTL), hypertensive and hyperglycemic animals (HT-DIAB), and hypertensive and hyperglycemic animals treated with macitentan (HT-DIAB+MAC25) for 60 days (final day of the experiment). B,C, and D, Quantitative analyses of immunoblot images were obtained by ImageJ software. Data are reported as means ± SE. The significance level for a null hypothesis was set at 5%. ^**^ *p* < 0.05compared to the HT-CTL 60 days group and ^¥¥^ *p* < 0.05 compared to the HT-DIAB 60 days group (ANOVA followed by the Tukey’s post-hoc test). N = 10 per group.

Figure 2 analyzes the RAS through protein expression and their respective graphical quantifications. In the HT-DIAB group, there was an increase in the production of angiotensin II. The HT-DIAB+MAC25 group, which had decreased ET-1 production, demonstrated an increase in the expression of the renin as well as angiotensin II.

**Figure 3:**
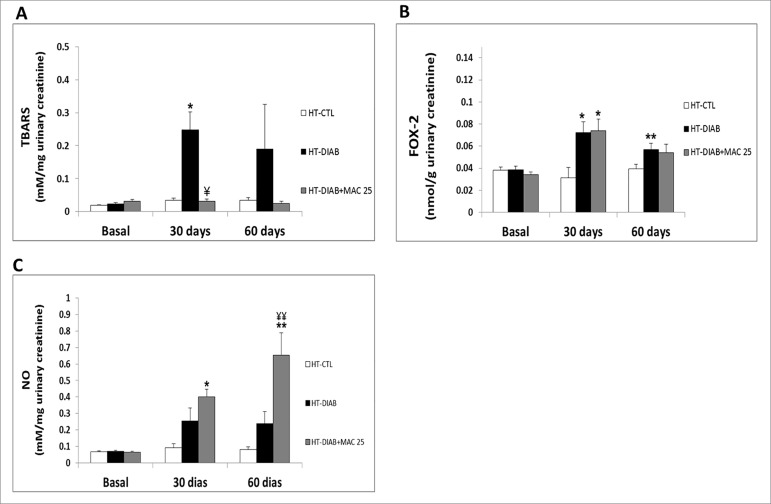
Analysis of the formation of reactive oxygen species and reactive nitrogen species. A, Quantitative analyses of thiobarbituric reactive substances (TBARS). B, Urinary hydrogen peroxide was measured by a modified ferrous ion oxidation xylenol orange version-2 (FOX-2). C, Nitric oxide (NO) in genetically hypertensive rats in the control group (HT-CTL), hypertensive and hyperglycemic animals (HT-DIAB), and hypertensive animals treated with Macitentan (HT-DIAB+MAC25) for basal, 30, and 60 days. Data are reported as means ± SE. The significance level for a null hypothesis was set at 5%. ^*^ *p* < 0.05 compared to the HT-CTL 30 days group; ¥P<0.05 compared to the HT-DIAB 30 days group, ^**^ *p* < 0.05 compared to the HT-CTL 60 days group, and ^¥¥^ p < 0.05 compared to the HT-DIAB 60 days group (ANOVA followed by the Tukey’s post-hoc test). N = 10 per group.

Figure 3 A shows the urinary levels of lipid peroxidation (TBARS). There was an increase in lipid peroxidation in the HT-DIAB group at 30 days when compared to their HT-CTL group. Conversely, in the HT-DIAB+MAC25 group, lipid peroxidation was not statistically different when compared to the HT-CTL group in all experimental periods.

Urinary hydroperoxides (Figure 3B) evaluated by FOX-2 increased in the HT-DIAB group at 30 and 60 days, but in the HT-DIAB+MAC25 group, an increase was seen only at 30 days. Additionally, at 60 days, the HT-DIAB+MAC25 group had similar lower levels when compared to the control rats. This result suggests, again, a potential antioxidant effect of macitentan.

Figure 3C analyzes urinary NO levels. Although there is a trend indicative of an increase, urinary NO levels in the HT-DIAB group were not statistically different when compared to those in the HT-CTL group at 30 and 60 days. However, the group treated with macitentan showed a significant increase in urinary NO concentration when compared to the HT-CTL group at 30 and 60 days. Since the renal injury determined by NGAL (Figure 1) decreased with the treatment of macitentan 25 mg/kg, the NO produced by the kidney may contribute to the amelioration of renal failure.

## DISCUSSION

Our experimental model utilized spontaneously hypertensive rats with concomitant hyperglycemia induced by streptozotocin. This model was chosen because the coexistence of DM and systemic arterial hypertension (SAH) increases the risk of developing chronic renal failure. This experimental model has characteristics very similar to diabetic renal injury found in humans.

We observed signs of diabetes including elevated glycemia, polyuria (due to hyperglycemic osmotic diuresis), and decreased body weight. In association with increased systolic blood pressure, these parameters lead to progressive loss of renal function. We identified proteinuria, increased sodium excretion, and increased creatinine and urea in hypertensive and hyperglycemic rats.

NGAL is a reliable diagnostic and prognostic biomarker for acute kidney injury (AKI) as its levels rise 2 h after the kidney injury. Moreover, studies have shown that NGAL levels in AKI and stable CKD groups are higher than those of control groups^34^^-^ 36, corroborating our results, since there was an increase in NGAL in the hypertensive and hyperglycemic rats at 60 days.

Recent studies have demonstrated the role of ET-1 in the initial phase of DN. Specifically, the expression of ET-1 receptors can be stimulated by hyperglycemia^37^. The kidney is an essential site of its production; ET-1 causes increased renal vascular resistance, reduced renal blood flow and glomerular filtration rate, and inhibition of salt and water reabsorption^37^.

At the same time, the RAS is also actively involved in the genesis and progression of DN, and there is already evidence of an interaction between ET-1 and other vasoactive substances, such as angiotensin II^38^.

Our results show that the kidneys of hypertensive and hyperglycemic rats exhibited stimulation of angiotensin II as well as ET-1, confirming the participation of these mediators of inflammation and endothelial dysfunction, respectively.

Oxidative and nitrosative stress play an important role in diabetes progression^39^^,^ 40. The hypertensive and hyperglycemic rats in our study showed an increase in the renal lipid peroxidation, urinary peroxides, and NO levels.

Since ET-1 plays an important role in the progression of diabetes, this study aimed to test a possible contribution of the effect of macitentan, which is a non-selective antagonist of ETA and ETB receptors and has received approval from the U.S. Food and Drug Administration (FDA) in 2014. However, other ET-1 selective antagonists have been used in the prevention of diabetic nephropathy, including use in clinical trials^22^. The macitentan was studied in hyperglycemia-induced kidney injury in mouse models of type 2 diabetes^41^, but inasmuch hypertension and diabetes are major risk factors for chronic kidney disease, our study evaluated the effect of ET-1 antagonist on renal function of rats simultaneously hypertensive and diabetic.

The dose of macitentan used in this study is consistent with that found in other studies^41^^,^ 42. Initially, we observed that hypertensive and hyperglycemic rats treated with 25 mg/kg of the antagonist exhibited neither a significant increase in the plasma concentration of creatinine and urea, nor an increase in the excretion of proteins. These results, together with the decrease in the expression of NGAL, suggest improvement in renal function.

ET-1 promotes natriuresis by acting on the collecting duct cells via ETB receptors^43^. However, we did not observe a significant decrease in sodium excretion in hypertensive and hyperglycemic rats treated with the antagonist macitentan. One hypothesis concerns the approximate 50-fold selectivity of macitentan for ETA receptors^42^, which would cause the effects of ET-1 mediated by the ETB receptor, such as natriuresis, to be at least partially preserved.

To confirm the effect of macitentan on the kidney, we analyzed the labeling for ET-1 and confirmed that there was a decrease in labeling in the renal tissue of hypertensive and hyperglycemic rats treated with macitentan. This result demonstrated that ET-1 receptor blockade decrease the production of this vasoconstriction agent.

Since ET-1 reduces the rate of glomerular filtration by promoting contraction of the afferent and efferent arterioles, ET-1 blockade may explain in part the improvement in renal function. However, in our experimental conditions, we observed that ET-1 receptor blockade increased the production of angiotensin II in renal tissue, which could counteract ET-1 receptor blockade by acting at its AT1 receptor, promoting arteriolar vasoconstriction.

Another hypothesis is that NO may act as a functional antagonist of angiotensin II, inhibiting vasoconstriction in glomerular arterioles^44^^,^ 45. The use of macitentan in hypertensive and hyperglycemic rats further increased urinary excretion of NO (165%). This increase in NO production may explain, at least in part, the improvement in kidney function indicated by the improvement in renal blood flow via the vasodilator effect of NO. It has already been shown in rabbit kidney that ET-1 stimulates the production of NO through the activation of ETB receptors^46^. Since macitentan is more selective for the ETA receptor, this increase in urinary output and excretion may be due to the action of the ETB receptor^42^^,^ 43.

Interestingly, the use of macitentan stimulated the expression of catalase, an important antioxidant enzyme. The increase in lipid peroxidation and urinary hydrogen peroxides was prevented by MAC treatment, suggesting that hydrogen peroxide was degraded by the catalase enzyme into water and oxygen^47^.

## CONCLUSION

We report that macitentan (25 mg/kg) inhibited the progression of renal injury induced by hyperglycemia and hypertension in rats, possibly by potentiating the antioxidant defense and preventing the increase of oxidative stress. A limitation of our work is the lack of nore specific markers of kidney function, but the markers used in the present study are those currently used in clinical practice. In conclusion, blocking the ET-1 receptor with macitentan may be an alternative in the prevention of DN in hyperglycemic and hypertensive rats. Intervention with macitentan should be confirmed in human clinical trials.
